# The Signaling Pathways Involved in the Antiatherosclerotic Effects Produced by Chinese Herbal Medicines

**DOI:** 10.1155/2018/5392375

**Published:** 2018-06-13

**Authors:** Li Lu, Xiaodong Sun, Yating Qin, Xiaomei Guo

**Affiliations:** ^1^Department of Cardiology, Tongji Hospital, Tongji Medical College, Huazhong University of Science and Technology, Wuhan 430030, China; ^2^Department of Anesthesiology, Puai Hospital, Tongji Medical College, Huazhong University of Science and Technology, Wuhan 430030, China

## Abstract

Cardiovascular diseases (CVDs) are considered to be the predominant cause of death in the world. Chinese herb medicines (CHMs) have been widely used for the treatment of CVDs in Asian countries for thousands of years. One reason of high efficacy of CHMs in treating CVDs is attributed to their inhibition in atherosclerosis (AS) development, a critical contributor to CVDs occurrence. Cumulative studies have demonstrated that CHMs alleviate atherogenesis via mediating pathophysiologic events involved in AS. However, there is deficiency in the summaries regarding antiatherogenic signal pathways regulated by CHMs. In this review, we focus on the signal cascades by which herb medicines and relevant extractives, derivatives, and patents improve proatherogenic processes including endothelium dysfunction, lipid accumulation, and inflammation. We mainly elaborate the CHMs-mediated signaling pathways in endothelial cells, macrophages, and vascular smooth muscle cells of each pathogenic event. Moreover, we briefly describe the other AS-related factors such as thrombosis, autophagy, immune response, and noncoding RNAs and effects of CHMs on them in the way of cascade regulation, which is helpful to further illustrate the molecular mechanisms of AS initiation and progression and discover newly effective agents for AS management.

## 1. Introduction

Cardiovascular diseases (CVDs) are the most common cause of health loss at home and abroad, by the fact that more than 13 million patients die from CVDs annually [1]. It is demonstrated that atherosclerosis (AS) is the pivotal pathological basis of CVDs. AS, characterized by formation of atherosclerotic plaques in the artery intima, could induce lumen stenosis or occlusion, finally leading to the occurrence of CVDs [2]. Thus, in order to reduce the prevalence of life-threatening CVDs, especially ischemic heart disease and stroke, the prevention and treatment of AS are of vital importance.

Over the past years, several drugs have been developed as therapeutic agents for AS and the representative one is the statin. However, there is evidence indicating that statin therapy is unable to decrease CVD risks in the majority of patients [3]. Moreover, liver dysfunction and myopathy, which are potentially adverse effects of statin application, make several patients stop receiving statin therapy, especially for those suffering hepatitis [4, 5]. It is urgent to explore alternative and complementary options with high efficiency and less side effects for AS management.

With a holistic and synergistic way, Chinese herbal medicines (CHMs) keep the balance of homeostasis in vivo. It is reported that a variety of herbal drugs and their extractives such as flavonoid, alkaloid, and terpenoid and patent products possess superior pharmacological properties in the prophylaxis and treatment of AS. Considering the effective clinical application of CHMs (Table 1), a plenty of studies have concentrated on the mechanisms of action underlying therapeutic effects for AS [6–8]. In this review, we will focus on the relevant signaling pathways modified by which CHMs exert beneficial effects in AS prevention and therapy.

## 2. Mechanisms of Action of CHMs

**Figure 1:**
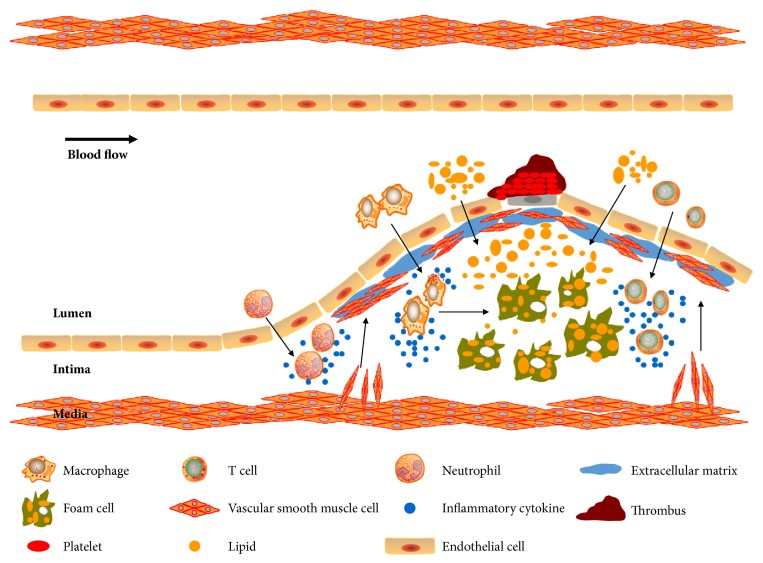
The pathogenesis of atherosclerosis. The endothelial dysfunction, inflammation, and lipid dysbolism induce excessive cholesterol disposition and leukocytes accumulation into the vascular intima. Then macrophages and neutrophil secret cytokines and T cells release immune regulatory factors, which promote AS progression. Moreover, macrophages uptake cholesterols and transform into foam cells, facilitating the formation of lipid core. VSMCs migrate to the subendothelium and release extracellular matrix, leading to the formation of fibrous cap and vascular remolding. In addition, platelets are activated and aggregate to the injured vascular endothelium, contributing to the thrombosis.

During the development of atheroma, multiple cells in the lesion environment including endothelial cells (ECs), macrophages, vascular smooth muscle cells (VSMCs), platelets, and lymphocytes are involved [9, 10] (Figure 1). CHMs have been shown to target specific signaling cascades in these cells to generate antiatherogenic effects; the detailed information will be discussed below.

### 2.1. Amelioration of Lipid Metabolism Disorder

#### 2.1.1. Triterpenoid

**Figure 2:**
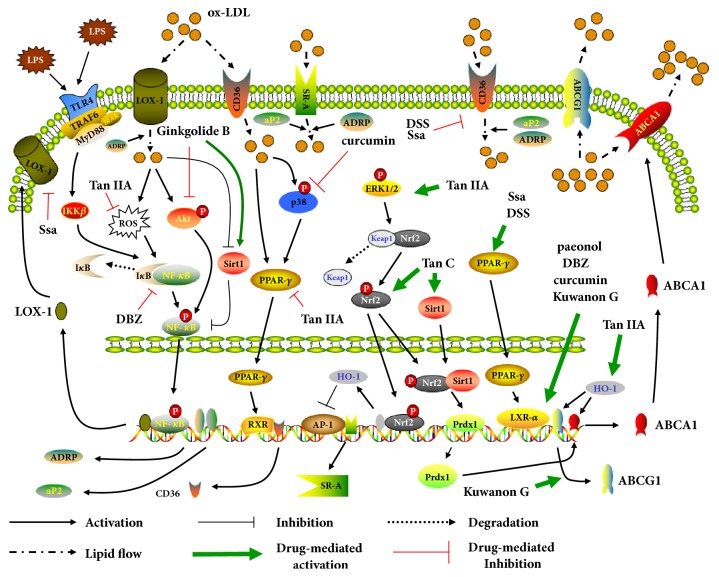
The signaling pathways by which CHMs alleviate lipid accumulation in macrophages.

Reverse cholesterol transport (RCT) is a cholesterol metabolism process through regulating the efflux of cholesterol from lipid-laden foam cells back to liver for recycling or excretion, which is mediated by signal molecules such as ATP-binding cassette transporters A1 and G1 (ABCA1 and ABCG1), peroxisome proliferator-activated receptor *γ* (PPAR-*γ*), and liver X receptor *α* (LXR-*α*) [11]. One pivotal antiatherogenic mechanism of Saikosaponin-a (Ssa) is attributable to the promotion of signal transduction of PPAR-*γ*/LXR-*α*/ABCA1 cascade, stimulating the outflow of cholesterol in macrophages [12] (Figure 2), while, with a LXR-*α* independent way, Tanshinone IIA (Tan IIA) increases the level of ABCA1 and ABCG1 by facilitating extracellular signal-regulated kinase (ERK)/nuclear factor-erythroid 2-related factor 2 (Nrf2)/heme oxygenase-1 (HO-1) axis [13]. In the presence of Tanshindiol C (Tan C), the content of lipids in macrophages stimulated by oxidized low density lipoprotein (ox-LDL) is markedly reduced, which is attributed to the drug-triggered activation of Nrf2 and Sirtuin 1 (SIRT1) and downstream peroxiredoxin 1/ABCA1 pathway [14].

Several studies report that PPAR-*γ* is response for cluster of differentiation (CD) 36 expression regulated by ox-LDL and Tan IIA inhibited cholesterol ingestion via suppressing PPAR-*γ* which transcriptional activates CD36 expression [15]. Moreover, ox-LDL uptake by lectin-like ox-LDL receptor-1 (LOX-1) induces production of reactive oxygen species (ROS) followed by nuclear factor *κ*B (NF-*κ*B) activation and subsequent LOX-1 expression, resulting in the positive feedback of cholesterol inflow in macrophages [16]. It is demonstrated that another mechanism underlying Tan IIA ameliorates atherogenesis to inhibit ox-LDL-triggered ROS/NF-*κ*B/LOX-1 loop [17]. Besides, ERK/Nrf2/HO-1 pathway activated by Tan IIA enables blocking the activity of activator protein-1 (AP-1) which mediates scavenger receptor-A (SR-A) expression, reducing SR-A-regulated cholesterol influx [13] (Figure 2).

It is documented that Tan IIA induces LDL receptor (LDL-R) production and increase LDL uptake in hepatic cells through stimulating sterol regulatory element-binding protein (SREBP) 2 pathway which mediates LDL-R expression and raising the nuclear Forkhead box O3a (FoxO3a) cascade which inhibits the expression of proprotein convertase subtilisin/kexin type 9 promoting LDL-R degradation [18].

#### 2.1.2. Phenolic Compound

Danshensu (DSS) could afford a cholesterol-lowing role in macrophages by virtue of stimulating the PPAR-*γ*/LXR-*α*/ABCA1 pathway [19]. Similarly, DSS derivative (DBZ) and paeonol ameliorate foam cell formation and enhance cholesterol efflux via activation of LXR-*α* and upregulation of ABCA1 [20, 21]. Moreover, it is proved that DBZ reduces foam cell formation via inhibiting macrophage lipid accumulation by suppressing Toll-like receptor 4 (TLR4)/NF-*κ*B/adipose differentiation-related protein (ADRP) cascade [22].

#### 2.1.3. Diarylheptanoid

By impeding the activation of ox-LDL evoked p38 MAPK (p38)/PPAR-*γ*/CD36 cascade, curcumin reveals similar effects in diminishing ox-LDL-upregulated CD36 level [23]. Liu et al. had shown that curcumin was a cholesterol efflux promoter through activating LXR-*α* and then ABCA1 upregulation [24].

#### 2.1.4. Alkaloid

Berberine (BBR), a kind of cholesterol-lowing herb extractive, activates ERK1/2 to stabilize LDL-R mRNA, leading to upregulation of LDL-R protein and decrease of serum LDL [25]. Additionally, various CHMs attenuate atheroma formation depending on blockade of triglyceride synthesis in hepatocytes. BBR and ginsenosides metabolite compound K (CK) have been proved to stimulate liver kinase B1/AMP-activated protein kinase (AMPK) signaling flow to phosphorylate acetyl-CoA carboxylase (ACC) and inhibit SREBP-1c/fatty acid synthase (FAS) axis, which followed by reduction of lipogenesis [26–28].

#### 2.1.5. Saponin

**Figure 3:**
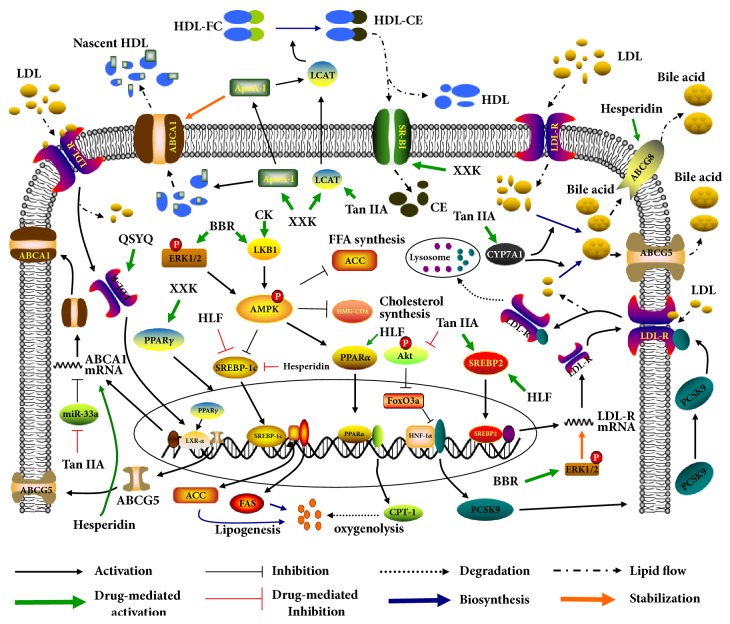
The pathway mechanisms underlying CHMs improve lipid metabolism in liver cells.

The liver exerts critical functions in the process of cholesterol synthesis and triglyceride generation and is the primary target organ of RCT. High-density lipoprotein (HDL), reversely associated with AS development, is response for transport of effluent cholesterol from peripheral tissues to the liver for removing. Di'ao Xinxuekang (XXK), saponin extractives of Dioscorea panthaica Prain et Burkill, is reported to enhance HDL generation by promoting PPAR-*γ*/LXR-*α*/ABCA1 pathway by which XXK improves the RCT process and alleviates atherosclerotic lesions [11] (Figure 3).

#### 2.1.6. Flavonoid

It has been confirmed that Kuwanon G is indicated to accelerate cholesterol elimination in macrophages by enhancing the signal transduction of LXR-*α*/ABCA1 cascade [29]. Dong et al. reported that the hawthorn leave flavonoids (HLF) triggered AMPK/PPAR-*α*/carnitine palmitoyl transferase 1 axis to increase the oxygenolysis of fatty acids, then reducing the generation of triglyceride.

Furthermore, cumulative data indicate that other CHMs improve dyslipidemia via diverse pathways. For instance, Qishen Yiqi pill (QSYQ) accelerates the excretion of bile acids to facilitate serum cholesterol uptake by liver via activating LDL-R/LXR-*α*/ABCG5 pathway [30].

### 2.2. Improvement of Cell Apoptosis

#### 2.2.1. Saponin

**Figure 4:**
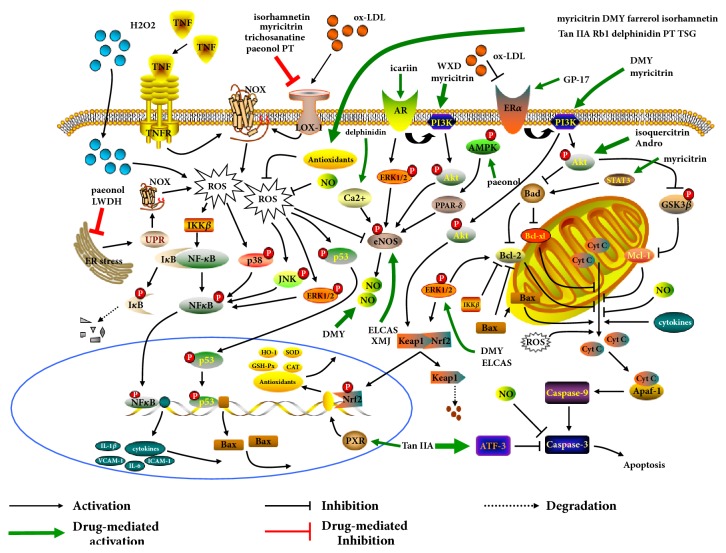
The effects at molecular level produced by CHMs in the attenuation of ECs apoptosis. ELCAS: Ligusticum chuanxiong and Angelica sinensis; XMJ: Xinmaijia; WXD: Wenxin decoction; and TSG: 2,3,5,4'-Tetrahydroxystilbene-2-o-*β*-D-glucoside.

Biological molecules including ox-LDL, TNF-*α*, and Ang II are the major driving forces in endothelial dysfunction linked to atherogenesis, through stimulating NADPH oxidase (NOX) and disrupting mitochondria respiration to generate excessive ROS [31]. Cellular analysis illuminates that ginsenoside Rb1 (Rb1) prevents ECs from TNF-*α*-induced insult via disturbing mitochondrial pathway of apoptosis, given that increment of Bcl-2/Bax ratio and caspase-3 evoked by TNF-*α*/c-Jun N-terminal kinase (JNK)/NF-*κ*B axis is inhibited by Rb1 [32] (Figure 4).

Considerable evidence has illustrated pivotal actions of PI3K/Akt cascade in cell survival. Gypenoside XVII (GP-17) is thought to directly suppress the apoptotic pathway by activating PI3K/Akt accompanied by Bad dysfunction [33].

#### 2.2.2. Flavonoid

It is demonstrated that p53 upregulated by ROS could aggravate mitochondrial apoptosis, because that p53 elevation facilitates Bax increase and Bcl-2 reduction. The protective ability of dihydromyricetin (DMY) against ECs apoptosis induced by H_2_O_2_ is partly attributed to inhibition of ROS-activated p53 and then improvement of imbalance of Bcl-2/Bax ratio, cyt-c release, and caspase-3 activation [34]. In addition, on stimulation of PI3K/Akt, isoquercitrin stimulates, resulting in GSK3*β* phosphorylation accompanied by Mcl-1 activation which blocks apoptosis of ECs [35]. Laboratory studies suggest that DMY, myricitrin, and GP-17 are able to alleviate ox-LDL-induced ECs apoptosis by activating PI3K/Akt/Nrf2/HO-1 pathway, which enhance intracellular antioxidative abilities to eliminate ROS [33, 36, 37]. Owing that endothelial NO synthase- (eNOS-) synthesized nitric oxide (NO) plays vital roles in maintaining the integrity of vascular endothelium, CHMs like icariin and Wenxin decoction (WXD) are found to phosphorylate eNOS and release NO by inducing PI3K/Akt signaling for mitigating atherogenic endothelial injury [38, 39].

Zeng et al. supported that ox-LDL maintained the survival of macrophages by upregulating antiapoptotic protein plasminogen activator inhibitor 2 (PAI-2) and apigenin exhibited proapoptotic effects on macrophages via inhibiting Akt/PAI-2 cascade [40]. However, there are clues showing that isohamnetin and Danshen granule are able to alleviate atheroma progression by inhibiting macrophages apoptosis via activating the PI3K/Akt/Nrf2/HO-1 axis [41, 42].

#### 2.2.3. Diterpenoid

Chen et al. showed that andrographolide (Andro) was capable of triggering PI3K/Akt and subsequent Bad inhibition, which impeded the activation of mitochondrial apoptotic pathway and then maintained the survival of ECs [43]. Additionally, in vitro studies report that Tan IIA and flavonoid myricitrin administration maintain the survival of ECs via retarding p53 expression, which hinder the pathway of H_2_O_2_/ROS/p53/caspase-3 [44, 45].

#### 2.2.4. Phenolic and Alkaloid

Paeonol and trichosanatine protect against ox-LDL-triggered ECs injury through abating LOX-1 expression followed by suppression of ox-LDL/LOX-1/ROS/p38 signal axis [46, 47]. One principle of paeonol and Liuwei Dihuang (LWDH) attenuated endothelial dysfunction is inhibiting ER stress, followed by decrease of unfolded protein response and restoration of C/EBP homologous protein and NAPDH level, resulting in reduction of ROS production [48–50]. Moreover, MEK/ERK1/2/eNOS and AMPK/PPAR-*δ*/eNOS cascades are also likely to be applied by herb drugs to produce NO, normalize ROS, and thus abolish oxidative stress-induce ECs apoptosis [38, 48].

### 2.3. Mediation of Cell Proliferation and Migration

#### 2.3.1. Saponin

Mounting data have confirmed the involvement of ECs in angiogenic process which is critical for accelerating AS development and exacerbating plaque vulnerability [51]. Antiangiogenic functions of CK are associated with activation suppression of p38 and Akt, which probably lead to decrease of proliferation proteins cyclin D1 and VEGF in ECs [52]. Yun et al. demonstrated that Panax notoginseng saponins (PNS) ameliorated plaque angiogenesis via reducing level of NOX4, resulting in decrement of ROS generation and subsequent VEGF expression [53].

#### 2.3.2. Diterpenoid

It is indicated that aberrant proliferation and migration of VSMCs aggravate atheroma progression and restenosis after balloon angioplasty [54]. Tan IIA has been reported to improve the activation of AMPK/p53/p21 axis to inhibit the expression of cyclin D1 stimulated by high glucose, finally alleviating the proliferation of VSMCs [55]. Wu et al. report that the antimigratory action of Tan IIA on VSMCs occurs by the increase of AMPK activity and subsequent block of NF-*κ*B cascade, which lower matrix metalloproteinase- (MMP-) 2 expression [55]. Additionally, suppressing TNF-*α*-activated MEK1/2/ERK1/2/AP-1 and Akt/IKK/NF-*κ*B cascade is another mechanism underlying Tan IIA reducing MMP-9 induction to decrease the movement of VSMCs [56]. Suh et al. offered evidence that cryptotanshinone (CTS) reduced MMP-9 level through inhibiting TNF-*α*-induced signal pathway involving in ERK1/2, p38, and JNK and then inactivation of AP-1 and NF-*κ*B [57].

Furthermore, there is evidence showing that Tan IIA is able to provide protective roles against atherogenesis by blocking ECs growth via disrupting the vascular endothelial growth factor (VEGF)/VEGF receptor 2 (VEGFR2) axis [58].

#### 2.3.3. Flavonoid

Dong et al. clarified that baicalin obviously inhibited platelet-derived growth factor- (PDGF-) stimulated proliferation of VSMCs, the mechanism of which was blocking signal pathway of PDGF receptor *β* (PDGFR*β*)/MEK/ERK1/2, followed by decrease of cyclin E and cyclin-dependent kinase 2 (CDK2) activity and increase of p27 level [59]. Icariin, hyperoside, and monoterpene compound paeoniflorin prevent VSMCs from ox-LDL-stimulated proliferation probably by abrogating LOX-1 expression, ROS generation, and ERK1/2 activation essential for mitogen-related cascade flow [60–62].

#### 2.3.4. Alkaloid

With the modulation of cascades linked to cell growth including Ras/Rac1, AMPK/p53/p21, and MEK/ERK1/2/Egr-1(c-fos), BBR lowers the expression of cell cycle-related proteins crucial for VSMCs proliferation [63, 64]. There are observations suggesting that the molecular basis for the antiproliferative effects of CHMs including alkaloid ligustrazine and flavonoid hydroxysafflor yellow A is that these herbs inhibit PDGF-induced activation of signal factors involved in p38, ERK1/2, and Akt pathway, leading to the expression depression of downstream cell cycle-associates molecules including cyclinD1, cyclinE, CDK2, and CDK4. In addition, ligustrazine and hydroxysafflor yellow A also enhance NO generation to elevate cGMP for decaying VSMCs growth, given the evidence of cGMP identified as a mitogenic suppressor [65, 66]

#### 2.3.5. Phenolic

Salvianolic acid B (Sal B) obviously attenuates LPS-modulated upregulation of MMP-2 and MMP-9, which might be ascribed to suppression of LPS-induced signaling of TLR4/MyD88, resulting in disorders of downstream pathways including ERK1/2, JNK, and COX-2 responsible for MMPs expression [67]. Moreover, blocking the proliferative signaling of protein kinase C (PKC)/Rac1/ROS and Ras/Raf/ERK1/2 is the potential mechanism for herb drugs like paeonol and isoflavonoid puerarin to extenuate diabetes-induced intimal hyperplasia [68, 69].

#### 2.3.6. Other Groups

Sparstolonin B, an isocoumarin compound, induces cell cycle arrest at G1 phase in ECs and significantly inhibits cell growth and vasculogenesis, and the mechanism might result from inactivation of NF-*κ*B and thereby decrease of cell cycle-promoting proteins [70]. Stimulating Nrf2-related pathway, antrodia salmonea enhances HO-1 and glutathione expression, favoring the scavenging of TNF-*α*-induced ROS, causing blockade of ROS-mediated I*κ*B kinase (IKK)/NF-*κ*B/MMP-9 signal transduction and thereby decrease of MMP-9 level [71]. In addition, curcumin, a kind of diarylheptanoid, has antimigratory roles in VSMCs by blocking MMP-9 and MMP-13 production in macrophages via suppressing AMPK/MAPKs and PKC cascade [72]. Moreover, Tongxinluo (TXL), widely used for treating CVDs, has been shown to reduce plaque burden and angiogenesis by decreasing angiogenic factor such as MMP-2 and VEGF through inactivating TNF-*α*/bone marrow kinase in chromosome X/NF-*κ*B/MAPKs pathway [73].

### 2.4. Inflammation Amelioration

#### 2.4.1. Diterpenoid

Upon induction of proinflammatory substances, the expressions of adhesion molecules and chemokines of ECs are elevated, favoring macrophages attachment and transmigration into subendothelium, then promoting AS lesions [9]. CTS encumbers expression of vascular cell adhesion molecule-1 (ICAM-1), intercellular adhesion molecule-1 (VCAM-1), and E-selection forced by ox-LDL on ECs via suppressing NOX4 to abrogate ox-LDL-induced ROS generation and sequential IKK*β* phosphorylation, I*κ*B degradation, and NF-*κ*B activation, and by restoring eNOS activity to produce NO [74]. Danshenol A and dihydrotanshinone I, other components isolated from Salvia miltiorrhiza Bunge, also lower macrophage adhesion to ECs triggered by LPS and TNF-*α* via hindering NOX4/IKK*β*/NF-*κ*B pathway [75, 76]. Moreover, Tan IIA perform antiatherogenic properties to lessen expression of adhesion molecules and chemokines in ECs, depending on modulating key signaling cascades containing Rho/Rho kinase, PI3K/Akt, Jak/STAT-3, and Wnt pathways [77–81].

In terms of inflammation attenuation of macrophages, CTS and Tan IIA are able to lessen synthesis and release of proinflammatory factors by mediating multitargets in pathways including TLR4/ IKK/NF-*κ*B and TLR4/MAPKs [82–85]. It is illustrated that compounds from Danshen could activate the cascade of PI3K/Akt/Nrf2/HO-1 in macrophages to enhance the generation of CO that weaken NF-*κ*B activity and AS development [86–88].

In VSMCs, Andro counteracts LPS/IFN-*γ*-elicited upregulation of iNOS and MMP-9; the mechanism underlying is the enhancement of nSMase/ceramide/PP2A cascade that abolish LPS/IFN-induced IKK/NF-*κ*B axis [89]. Moreover, Chen et al. showed that the relief of TNF-*α*-stimulated NF-*κ*B activation and then iNOS expression was due to Andro-regulated inflammation restriction in VSMCs by virtue of inhibiting JNK/Akt pathway [90].

#### 2.4.2. Saponin

Ginsenoside F1 could upregulate zinc finger protein A20 to weaken ox-LDL-elicited LOX-1/ROS/NF-*κ*B pathway for lowering levels of ICAM-1, MIP-1*δ*, and IL-1*α* [91]. The inducible expression of VCAM-1 in response to ox-LDL is abolished in ECs with PNS and Rb1 preconditioning; this effect is primarily due to activation of Nrf2 followed by elevation of HO-1 and superoxide dismutase, causing intervention of ROS/TNF-*α*/p38 cascade [92]. Yu et al. demonstrated that the therapeutic utility of XXK in AS plaques had been partially ascribed to the anti-inflammatory ability of blocking transduction of TLR4/MyD88/IKK/NF-*κ*B cascade in macrophages [93]. Moreover, Diosgenin preconditioning diminishes level of adhesion molecules in VSMCs exposed to TNF-*α* via restraining the ROS/MAPKs and ROS/Akt pathway and downstream NF-*κ*B activation [94].

#### 2.4.3. Flavonoid

It is deciphered that quercetin has pharmacological efficacies on diminishing level of proinflammatory cytokines through prohibiting TLR/NF-*κ*B signal pathway in ECs [95]. Wogonin and Bushenningxin decoction have been indicated to activate the estrogen receptors on ECs to upregulate eNOS expression and heighten NO synthesis, leading to blockade of proinflammatory NF-*κ*B signaling [96, 97]. Additionally, Lee et al. reported that wogonin was effective in ameliorating inflammation state in macrophages by suppressing Ca^2+^/STAT signal axis in macrophages, as seen by decrease of cytokines including IL-1*β*, MCP-1, and MIP-1*α* [98]. Disrupting transcription activity of p50-p65 heterodimer, procyanidins decline macrophage-produced IL-6 and COX-2 via blocking LPS-initiated TLR4/NF-*κ*B pathway [99]. Ingredients in dracocephalum moldavica normalize the levels of VCAM-1 and ICAM-1 in VSMCs by suppressing TNF-*α*-triggered NF-*κ*B signaling [100]. Moreover, icariin reverses LPS-induced NF-*κ*B activation and cytokines production in macrophages via boosting PI3K/Akt pathway, whereas saponin glycyrrhizic acid and triterpenoid Ssa restrain inflammation by encumbering PI3K/Akt cascade [12, 101, 102]. This discrepancy might be attributed to the complex of signal networks and the difference of compound category, which is needed to be further elucidated.

#### 2.4.4. Alkaloid

The reduced attachment of macrophages to ECs after pretreatment with BBR is due to diminution of ROS-inducible adhesion molecules expression caused by AMPK/nuclear respiratory factor 1/uncoupling protein 2 axis [103]. Wu et al. illustrated that the revulsive expression of IL-6 and iNOS on stimulation with LPS was abolished in macrophages with coptisine coincubation, and the mechanism underlying this effect results from inhibiting MAPKs and Akt signaling and subsequent NF-*κ*B activation [104].

#### 2.4.5. Isoflavonoid

Biochanin-A, a bioactive isoflavonoid, is shown to reduce LPS-elicited TNF-*α*, IL-1*β*, and IL-6 expression in macrophages through inhibiting TLR4-dependent p38/ATF2 and NIK/IKK/NF-*κ*B pathway [105]. Furthermore, red clover extracts-enhanced PPAR-*α* and BBR-activated PPAR-*γ* have the similar function in terminating transduction of inflammatory pathways, seeing that signaling of PPAR-*α* and PPAR-*γ* abolishes I*κ*B degradation, NF-*κ*B activation, and binding to DNA regions in macrophages [106, 107].

#### 2.4.6. Monoterpenoid and Triterpenoid

The decrease of IL-6, IL-1*β*, and TNF-*α* production in VSMCs after paeoniflorin treatment is ascribed to alleviation of TLR4/MyD88/NF-*κ*B cascade [108]. Gu and colleagues found that celastrol reduced macrophages expression of iNOS, TNF-*α*, and IL-6 via mitigating ox-LDL-evoked LOX-1/NOX/ROS/NF-*κ*B cascade [109].

#### 2.4.7. Another Class

Capsaicin augments Ca2^+^ dependent PI3K/Akt/eNOS pathway to boost NO generation, which maintain the stability of I*κ*B and combination with NF-*κ*B upon LPS stimulation, leading to levels of ICAM-1, VCAM-1, and MCP-1 back to normal [110]. Meng et al. reported that curcumin exhibited anti-inflammatory actions in VSMCs by abating LPS-provoked TLR4/NOX/ROS cascade accompanied by blockade of ERK1/2 and p38 signaling and NF-*κ*B inactivation, as explained by decrease of MCP-1 and TNF-*α* [111].

Daotan decoction has been corroborated to counteract TNF-*α*-induced ICAM-1 on ECs via multipathway mode, evidenced by encumbrance of p53/p21, JNK, and p38 cascades [112, 113]. Zheng et al. manifested that Longxuetongluo capsule played atheroprotective roles by curtailing contents of COX-2, VCAM-1, and MCP-1 via restraining ox-LDL-provoked ERK1/2(p38)/IKK/NF-*κ*B signal [114]. Sun et al. showed that Tianxiangdan granule afforded antiatherogenic actions by inhibiting inflammatory p38/NF-*κ*B signal pathway in ApoE-/- mice [115]. With the encumbrance of IRS-1/PI3K/Akt signal flow, Shenyuandan capsule blocks activation of NF-*κ*B and then expression of IL-6 and TNF-*α* in aortas [116]. Thus, the above herb drugs execute anti-AS roles through diverse signal pathways, and NF-*κ*B represents the convergence of most of these cascades, hinting that NF-*κ*B acts as a pivotal target of CHMs for AS management.

### 2.5. Alleviation of Thrombogenesis

#### 2.5.1. Terpenoid

As normal blood flow is decided by platelets, clotting factors, and fibrinolytic molecules, dysfunction of them could facilitate thrombus formation in the site of plaque lesions, contributing to atherogenesis and related complications occurrence. Fu and colleagues find that triterpenoid substances from callicarpa nudiflora hook have antiplatelet roles by eliminating ADP and TXA_2_-induced platelet activation and aggregation via inhibiting PI3K/Akt/GSK3*β* and RhoA pathway, respectively [117] (Table 2). Chen et al. provided initial proof that Atractylodes lactone compounds were antithrombotic because of their effects on lessening platelet accumulation and secretion triggered by agonists via blocking p38 and Akt signaling [118]. After Andro administration, collagen-induced platelet activation is restrained, the mechanism is Andro-regulated augmentation of eNOS/NO/sGC signal flow, which in turn catalyze synthesis of cGMP required for reducing activities of p38, IKK*β*, PI3K, and PLC*γ*2 implicated in the process of collagen-regulated Ca^2+^ elevation [119, 120]. Investigation into mechanisms reveals that Tan IIA suppresses platelet activation and TF expression via mediating multiple pathways including ER*α*/PI3K/Akt, ROS/NF-*κ*B, and ERK-2 [121–123]. Moreover, *β*-Elemene, which belongs to sesquiterpenoid, is indicated to induce the PI3K/Akt/eNOS pathway to increase NO level, thereby suppressing platelet activation and aggregation [124].

#### 2.5.2. Saponin

Given that inflammatory factors are contributors to upregulation of TF for accelerating AS development, gypenoside XLIX and extracts of red yeast rice restore TF level and weaken AS progression via impairing NOX/ERK1/2/NF-*κ*B and boosting PPAR-*α* pathway, respectively [125, 126]. Pan et al. stated that Rb1 and Rg1 encumbered platelet accumulation and thrombosis via increasing NO synthesis by triggering PI3K/Akt and CAT-1/L-arginine cascade [127].

#### 2.5.3. Other Compounds

Other CHMs like lignin Gomisin J, flavonoid rumex acetose L, and isoflavonoid puerarin also enhance eNOS activity and NO generation by means of mediating diverse cascades including Ca^2+^/CaMI, ER/PI3K/Akt, and CaMKII/AMPK, indicating their implication in encumbering thrombosis and AS progression [128–130]. Sal B is shown to prevent TNF-*α*-dependent ERK1/2/AP-1 and NF-*κ*B cascade, leading to decrease of PAI-1 level and restoration of malfunction of fibrinolytic system [131].

### 2.6. Improvement of Autophagy

#### 2.6.1. Flavonoid

It is well established that autophagy exerts vital roles in regulating endothelial function, macrophage lipid metabolism, VSMCs phenotypic conversion, thrombosis, and angiogenesis which are involved in atheroma development [132, 133]. Jin et al. provided evidence that enhanced autophagy, resulting from AMPK/SIRT1 signal pathway induced by delphinidin-3-glucoside, attenuated ox-LDL-elicited injury in ECs [134] (Table 3). Gossypetin, a type of flavonoid, effectively weakens ox-LDL-caused ECs damage and this phenomenon is explained by drug-mediated inhibition of class I PI3K/Akt cascade and activation of class III PI3K/Beclin-1/microtubule associated protein light chain 3 (LC3) pathway, thus leading to upregulation of autophagy [135].

#### 2.6.2. Stilbenoid

The involvement of autophagy in pterostilbene- (PT-) mitigated ECs apoptosis is crucial, because that Ca^2+^/CaMKK*β*/AMPK pathway induced by PT reduces TUNEL labeled cells [136]. Resveratrol, classified as a autophagy activator, is capable of boosting autophagic processes to ameliorate inflammation and injury in ECs elicited by TNF-*α* and ox-LDL, and the mechanism is attributed to accentuation of cAMP/AMPK/SIRT1 cascade followed by elevation of LC3II and reduction of p62 [137, 138].

#### 2.6.3. Alkaloid and Saponin

BBR is identified to suppress level of inflammatory factors in macrophages by activating AMPK, which blocks the autophagy inhibitor mammalian target of rapamycin (mTOR), leading to initiation of autophagy responsible for inhibiting NF-*κ*B activity [139]. Inducing ROS to restrain PI3K/Akt/mTOR cascade, BBR-mediated sonodynamic therapeutics contribute to the autophagic processes which raise ABCA1 expression, favoring the inhibition of cholesterol uptake in macrophages [140]. Furthermore, ECs apoptosis promoted by ox-LDL is abolished in the presence of increase of Beclin-1 and LC3II modulated by elatoside C-induced FoxO1 overexpression [141]. With autophagy activation, celosin and polyphenolic luteolin also alleviate lipid accumulation in macrophages and then restrain AS expansion [142, 143].

### 2.7. Other Related Mechanisms

#### 2.7.1. Modulation of Immune Response

Cumulative papers attempt to comprehend how immune system participates in the pathogenic processes of atherogenesis, as innate and adaptive immunity are validated to be correlated with all stages of AS [144]. It is confirmed that some CHMs impede atherogenesis via mediating immune response. For example, TXL is shown to induce the regression of AS, at least partly via inhibiting ox-LDL-evoked DCs maturation, as illustrated by reduction of membrane CD40, CD86, and CD1a [145]. Moreover, one property of baicalin and geniposide ameliorating AS is ascribed to the gathering blockade of DCs in plaque areas that launch proatherogenic immune reactions [146]. Owing that regulatory T cells (Tregs) hinder T helper (Th) cells-induced inflammation, QSYQ and amygdalin increase the content of Tregs in vascular lesions which delay the development of AS [30, 147]. Additionally, QSYQ directly inhibit Th17 cells in AS areas and then lower the release of IL-17, a proatherogenic cytokine [30].

#### 2.7.2. Regulation of Noncoding RNAs

Noncoding RNAs (ncRNAs), a group of RNA molecules mainly containing long noncoding RNAs (lncRNAs) and microRNAs (miRNAs), are capable of affecting pathogenic processes of AS by mediating lipid metabolism, cellular apoptosis and proliferation, inflammations, etc. [148, 149]. Tan IIA accelerates the clearance of cholesterol in the vasculature by abolishing HFD-evoked miR-33a expression to elevate ABCA1 level in the liver, resulting in upregulation of HDL secretion and RCT pathway [150]. Genipin impedes lipid deposition via boosting level of miR-142a-5p, which in turn lessen the lipogenesis pathway of SREBP-1c/ACC(FAS) in hepatocytes [151]. Following paeonol treatment, a decrease in ECs apoptosis and TNF-*α* production is seen, probably explained by paeonol-mediated suppression of miR-21/TNF-*α* axis and subsequent apoptotic cascade [152]. Moreover, TXL mitigates the inflammation via triggering Akt accompanied by reduction of miR-155 and then TNF-*α* expression [153]. LncRNA TUG1 overexpression is implicated in ECs apoptosis induced by ox-LDL, and tanshinol improves ECs damage via reducing TUG1 level which is followed by upregulation of miR-26a and decrease of TRPC6 responsible for calcium overload [154]. In AngII-stimulated ApoE-/- mice, Xiaoxianggou administration rescue miR-203 downregulation to reduce generation of Ets-2 which potentiates angiogenesis and autoimmunity, causing the regression of AS plaques [155].

## 3. Conclusions

**Figure 5:**
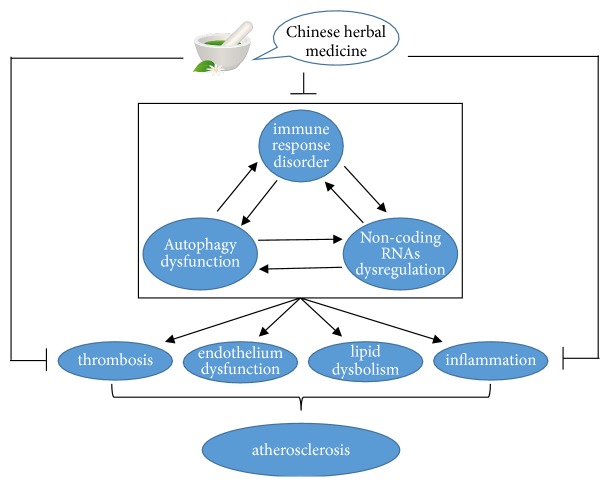
The schematic flowchart of diverse pathogenic mechanisms of AS and the intervention exerted by CHMs.

As a representative of complementary and alternative medicine, CHMs have been prescribed to patients for thousands of years in Asian countries for preventing and treating diseases. In this review, we place the emphasis on the signal pathways by which CHMs produce antiatherogenic functions. We find that several herb medicines could regulate one signal pathway to provide multiple roles against AS and an herb drug is able to exhibit one anti-AS action by mediating two or more cascades, suggesting the pleiotropic and multitargeted effects of CHMs in AS alleviation. Besides, apart from directly improving the cascades of lipid dysbolism, endothelium injury, and inflammation response, herb drugs afford atheroprotective actions through mediating the processes of thrombosis, autophagy, immune reaction, and ncRNAs expression, majority of which converge on the pathways of the above three AS contributors (Figure 5). However, there are several limitations existing in the research field of CHMs which we cannot ignore. At first, the counterevidence proving the anti-AS roles of CHMs-induced signal pathways is deficient in several literatures, especially in animal studies. So, rigorous logical thinking and experimental design are recommended. Moreover, most of the CHMs-related studies are published in Chinese journals and the theories of ancient traditional Chinese medicine are obscure to the western world, both of which impede the development of CHM research field in the globe. Thus, it is imperative to present more pharmacological and therapeutic findings of CHMs to the international anti-AS study organization and use modern scientific ways to clarify the theory of CHM. Additionally, the majority of the herb drugs like Tan IIA, icariin, BBR, paeonol, and curcumin have been proved to be effective in suppressing AS progression by preclinical experiments, but relevant clinical trials to investigate the safety and effectiveness are scarce. So, it is urgent to perform plentiful well-designed clinical studies with standard and strict procedures to afford reliable and sufficient evidences for the clinical application of these herb drugs. Furthermore, some patent drugs such as Shexiang Baoxin pill have been widely used to treat CVDs in the clinic, whereas the underlying therapeutic mechanisms are poorly understood [156]. Uncovering the antiatherogenic mechanisms of these medicines is helpful to enhance the theoretical basis of their clinic application. In addition, with the development of modern biological technology like bioinformatics analysis and network pharmacology, more and more bioactive compounds from herbs and relevant signal pathways offering protective roles against AS will be discovered.

## Figures and Tables

**Table 1:** The classification of compounds from CHMs with anti-AS roles.

Category	Compound
Terpenoid	Saikosaponin-a, tanshinone IIA, tanshindiol C, ginkgolide B, andrographolide, paeoniflorin, cryptotanshinone, danshenol A, dihydrotanshinone I, celastrol, 1, 6-di-O-caffeoyl-*β*-D-glucopyranoside, atractylenolide, *β*-Elemene
Saponin	Xinxuekang, compound K, ginsenoside Rb1, gypenoside XVII, Ginsenoside F1, glycyrrhizic acid, Diosgenin, Elatoside C, Celosin
Alkaloid	Berberine, trichosanatine, ligustrazine, coptisine,
Flavonoid	Kuwanon G, myricitrin, dihydromyricetin, isoquercitrin, icariin, apigenin, isohamnetin, baicalin, hydroxysafflor yellow A, hyperoside, quercetin, wogonin, procyanidin, dracocephalum moldavica, rumex acetose L, delphinidin-3-glucoside, gossypetin
Isoflavonoid	Puerarin, Biochanin-A
Phenolic	Danshensu, paeonol, salvianolic acid B, luteolin
Stilbenoid	Pterostilbene, resveratrol
Iridoid	Geniposide, Genipin
Diarylheptanoid	Curcumin

**Table 2:** The signal pathways underlying CHMs inhibit the thrombosis.

Ingredient	Herb medicine	Object	Stimulus	Role	related pathway
1, 6-di-O-caffeoyl-*β*-D-glucopyranoside	Callicarpa nudiflora Hook	Platelet	ADP, AA	*α*IIb*β*3, 5-HT, TXA2, platelet aggregation↓	PI3K/Akt/GSK3*β*, RhoA
Atractylenolide	Atractylodes macrocephala	Platelet	ADP, collagen, thrombin	platelet aggregation and secretion↓	p38, PI3K/Akt
Andrographolide	Andrographis paniculata	Platelet	Collagen	platelet aggregation, Ca2+, TxB2↓	eNOS/NO/sGC/cGMP, PI3K/Akt/p38/cPLA2, PLC*γ*2/DAG/PKC
				platelet aggregation, Ca2+↓	eNOS/NO/sGC/cGMP, p38/ROS/IKK*β*/NF-*κ*B/ERK2
Gomisin J	Schisandra chinensis	EC	None	eNOS, NO↑	Ca2+/CaMI, PI3K/Akt
*β*-Elemene	Curcuma Wenyujin	EC	None	eNOS, NO↑	PI3K/Akt
Puerarin	Pueraria lobata	EC	TNF-*α*	eNOS, NO↑	ER/PI3K/Akt, CaMKII/AMPK
Tanshinone IIA	Salvia miltiorrhiza Bunge	Macrophage	Ox-LDL	TF↓	ROS/NF-*κ*B
		Platelet	None	Platelet activation↓	ER*α*/PI3K/Akt
		Platelet	ADP	Platelet activation↓	ERK-2
Xuezhikang	Red yeast rice	Macrophage	Ox-LDL	TF↓, SOD↑	NOX/ROS/ERK1/2/NF-*κ*B
Gypenoside XLIX	Gynostemma pentaphyllum	Macrophage	LPS	TF↓	PPAR-*α*
Salvianolic acid B	Salvia miltiorrhiza Bunge	EC	TNF-*α*	PAI-1↓	ERK1/2/AP-1 (NF-*κ*B)

**Table 3:** The signal pathways responsible for CHMs-induced regulation of autophagic processes.

Agent	Herb medicine	Object	Stimulus	Role	related pathway
Delphinidin-3-glucoside	Grape seed	EC	Ox-LDL	Cell viability ↑, apoptosis↓; LC3II↑, p62↓	AMPK/SIRT1
Gossypetin	Hibiscus	EC	Ox-LDL	LDH, cleaved caspase-3 and PARP-1↓; LC3II and Beclin-1↑, p62↓	PTEN/class I PI3k/Akt, class III PI3K/Beclin-1
Pterostilbene	Blueberry	EC	Ox-LDL	TUNEL-positive cell↓; LC3II↑, p62↓	Ca^2+^/CaMKK*β*/AMPK/mTOR
Resveratrol	Grape	EC	TNF-*α*	ICAM-1, COX-2, MMP-9↓; LC3II↑, p62↓	ATP/cAMP/AMPK/SIRT1
		EC	Ox-LDL	Cell vability and SOD↑; LC3II/LC3I↑, p62↓	AMPK/SIRT1
Elatoside C	Aralia elata Seem	EC	Ox-LDL	TUNEL-positive nuclei, Bax, caspase-9 and -3, ROS↓, Bcl-2↑; LC3II and Beclin-1↑, p62↓	FoxO1/Beclin-1, LOX-1/NOX/ROS/Caspase
Berberine	Coptis chinensis	macrophage	Ox-LDL	MIP-1*α*, RANTES↓, IL-10↑; LC3II/LC3I↑, p62↓	AMPK/mTOR
			None	ABCA1, ROS↑; LC3II/LC3I↑, p62↓	PI3k/Akt/mTOR
Arglabin	Artemisia glabella	macrophage	LPS	IL-1*β*, IL-18↓; LC3II↑	unknown
Celosins	Celosia argentea L.	macrophage	Ox-LDL	CD36, SR-A1↓, ABCA1, ABCG1↑; LC3II/LC3I↑, Beclin-1↑	unknown
